# Activities-Specific Balance Confidence in People with Multiple Sclerosis

**DOI:** 10.1155/2012/613925

**Published:** 2012-08-07

**Authors:** Ylva Nilsagård, Anna Carling, Anette Forsberg

**Affiliations:** ^1^Centre for Health Care Sciences, Örebro County Council, P.O. Box 1324, 701 13 Örebro, Sweden; ^2^School of Health and Medical Sciences, Örebro University, Sweden; ^3^Department of Physiotherapy, Örebro University Hospital, 701 85 Örebro, Sweden; ^4^Family Medicine Research Centre, Örebro County Council, P.O. Box 1613, 701 16 Örebro, Sweden

## Abstract

*Objective*. To evaluate the validity of the Activities-specific Balance Confidence scale (ABC) in people with multiple sclerosis (PwMS). *Design*. A multicentre, cross-sectional study. *Setting*. Six rural and urban Swedish sites, including specialized units at hospitals and primary care centers. *Participants*. A sample of 84 PwMS with subjective gait and balance impairment but still able to walk 100 m (comparable with EDSS 1–6). *Outcome Measures*. Timed Up and Go, Timed Up and Go^cog^, 25-foot Timed Walk Test, Four Square Step Test, Dynamic Gait Index, Chair Stand Test, 12-item MS Walking Scale, self-reported falls, and use of assistive walking device were used for validation. *Results*. The concurrent convergent validity was moderate to good (0.50 to −0.75) with the highest correlation found for the 12-item MS Walking Scale. The ABC discriminated between multiple fallers and nonfallers but not between men and women. Ecological validity is suggested since ABC discriminated between users of assistive walking device and nonusers. The internal consistency was high at *α* = 0.95, and interitem correlations were between 0.30 and 0.83. *Conclusion*. This study supports the validity of the ABC for persons with mild-to-moderate MS. The participants lacked balance confidence in many everyday activities, likely restricting their participation in society.

## 1. Introduction

Multiple sclerosis (MS) is the most common progressive neurological disease among younger adults, with approximately 17000 people diagnosed in Sweden in 2011 [1] and an estimated 2.5 million people with MS (PwMS) worldwide [2]. More women than men are diagnosed as having MS; the ratio is at least 2 : 1 [3]. Balance impairments are common [4], and knowledge of the underlying causes is increasing [5]. Imbalance is reported as an initial symptom [6] and is present in minimally impaired PwMS [7] and even in clinically isolated syndrome suggestive of MS [8]. Imbalance increases the risk of falls; the prevalence of falls has been reported as being between 48% (EDSS not reported; eligibility criteria: able to walk independently or with a can, without relevant cognitive and orthopedic impairments) and 63% (EDSS 3.5–6.0) [9–12]. There is no consensus regarding differences in the risk of falls between men and women with MS. Finlayson et al. reported that men have a greater fall risk than women [10], but a study of 76 PwMS did not confirm this result [12]. 

The complexity of imbalance can be explained by the multiplicity of control systems involved [13]. The actual performance of balance-demanding activities also includes a psychological component. Studies investigating the psychological aspects of imbalance in PwMS have primarily used the concept “fear of falling” [14, 15], which is defined as a lasting concern about falling that can lead to curtailed activity [16]. Self-efficacy is defined as a person's confidence in being able to perform a given task. High self-efficacy increases the chance of succeeding in performing an activity, regardless of actual physical ability [17, 18]. PwMS who report fear of falling have been shown to have a greater risk for falls [10], and being concerned about falling is common in PwMS, whether or not they have recently experienced a fall [19]. A majority of those who were concerned about falling also reported having limited their activity as a consequence of their concern. If a person is not confident in maintaining their balance, they are unlikely to actually perform activities within their capacity. Strengthening the patient's confidence is just as important as improving function, and it is essential for caregivers to be aware of how confident a person is in managing different activities. It is possible to strengthen a person's balance confidence via interventions [20].

One measure of balance confidence is the Activities-specific Balance Confidence (ABC) Scale. The ABC scale was initially designed for elderly people, as a development of the Falls Efficacy Scale [21] and has captured interest worldwide and has been translated into several languages [22–30], and some cultural adaptations have been made. Besides cultural aspects, environmental aspects as climate must be considered when applying the scale. 

It is possible that men and women differ in terms of balance confidence, but the evidence is inconclusive. In a sample of older adults (*n* = 475), men scored significantly higher balance confidence than women (*P* < .002) [31], while in another sample (*n* = 60), no gender difference was found [32]. Middle-aged and older women with MS are more likely to report fear of falling than men with MS [14] when investigated with an interview question (yes/no). It has not yet been shown whether or not this result can be generalized to other samples of PwMS.

The ABC scale has been used for PwMS and validated once (*n* = 51) using an Italian translation [28]. Convergent validity for the clinically assessed measures was *r* = 0.54 for the Dynamic Gait Index (DGI), *r* = 0.48 for the Berg Balance Scale (BBS), and *r* = −.38 for the Timed Up and Go test (TUG). The ABC discriminated between fallers and non-fallers and was slightly better at identifying non-fallers (specificity = 77%) than fallers (sensitivity = 74%). Retrospective falls only one month before the assessment procedure were used to define a faller, and people with primary progressive MS was excluded. No analyses were reported for potential differences between men and women. The validity of ABC for PwMS seems promising, but the results need to be verified in larger samples also including people with primary progressive MS and in different settings. Extending the period for self-reported falls may give a more reliable result. In the present study, a Swedish sample with mild-to-moderate MS was used, and falls were reported for two months. 

There is no gold standard for measuring balance confidence. In the present study, the TUG and the DGI were included to allow comparison of the results with the work by Cattaneo et al. [28]. Additional outcome measures were also used, reflecting gait speed, dual tasks, stepping in different directions, the time taken to repeatedly rise to standing and sit down again, and perceived limitation of walking due to MS. The hypothesis was that the concurrent convergent and interitem validity would be moderate to good and that the ABC could discriminate for fallers and users of assistive walking device but not for gender. 

The aim of this study was to evaluate concurrent convergent validity, discriminative validity, interitem validity, and internal consistency of the ABC of people with mild-to-moderate MS. 

## 2. Methods

### 2.1. Design

A multicentre, cross-sectional data collection was conducted from September 2010 to June 2011 at six sites from both rural and urban areas in Sweden representing four county councils (Örebro, Sörmland, Värmland, and Västmanland). 

### 2.2. Participants

The study included data from 84 persons with mild-to-moderate MS diagnosed according to the McDonald criteria [33]. Data were obtained from the first measure point in a randomized controlled trial evaluating balance exercise [34]. The inclusion criteria were self-perceived impaired gait and balance ability in standing and walking activities, and remaining ability to walk at least 100 m without rest but with the use of assistive device if needed (comparable with EDSS 1–6). The exclusion criteria were inability to understand the instructions or fill in the rating scales, ongoing exacerbation affecting balance, or other disease preventing them from performing the measurements. The study followed the Helsinki declaration and was approved by the Regional Ethics Committee (Dnr 2010/263). 

### 2.3. Procedure

Data were collected at one time point at the respective departments of physiotherapy by physiotherapists trained in securing according to test protocol. The tests were performed in a standardized order as described below. The physiotherapist was present and available for questions when the rating scales were filled in. 

### 2.4. Outcome Measures

The ABC scale measures balance confidence while performing 16 different activities [35] incorporating static, dynamic, proactive, and reactive balance [36]. 

The Timed Up and Go test (TUG) is a commonly used instrument which measures the time taken to rise from a chair, walk 3 m, turn, walk back, and sit down again [37]. It is valid [28] and reliable for PwMS, and one measurement is sufficient [38]. In the present study, the participants were encouraged to walk safely but as quickly as possible. One practice attempt was allowed before the actual testing.

The TUG^cognitive^ is performed by adding a cognitive component to the TUG (backward counting in multiples of three) [39]. Those reporting falls tend to take longer on this test compared to non-fallers [12]. One attempt was recorded. 

The 25-foot timed walk test (25TWT) measures walking speed [40]. The 25TWT is part of the MS Functional Composite disability assessment and is considered valid and reliable for PwMS also as an individual test [40–42]. The test was repeated twice, and the mean value was used in further calculations. 

The Dynamic Gait Index (DGI) consists of eight walking items involving actions such as changing speed, moving one's head, turning, stepping over or walking around obstacles, and climbing stairs [36]. Performance on each item is rated between 0 and 3 by the physiotherapist, with a higher score indicating better performance. The DGI is valid, and the interrater reliability is good when used among PwMS [43].

The Four Square Step test (FSST) measures the ability to step over four 2.5 cm height sticks (forwards, sideways, backwards, and sideways) first clockwise and then back again, while facing forwards [44]. It was originally designed for older people but has also been used for PwMS [12].

The Chair Stand Test (CST) is a measure of functional strength and balance in which the time is registered for a sequence of 10 sit-to-stands from a chair [45]. The use of armrests was allowed.

Fall was reported retrospectively for two months prior to data collection and was defined as an unexpected contact of any part of the body with the ground. Falls were categorized as no fall, one fall, or two or more falls (multiple) falls. Data on assistive walking device indoors and outdoors was collected. 

### 2.5. Statistical Procedure

Nonparametric methods were used due to data distribution. Concurrent convergent validity between the ABC scale and the other measures and interitem correlation were estimated using Spearman's rho. Internal consistency was estimated using Cronbach's alpha. Differences between men/women, single or multiple fallers/non-fallers, and use of assistive walking device or not were calculated using the Mann-Whitney *U*-test (two-tailed). The significance level was set at *P* ≤ 0.05. 

## 3. Results

Sixty-four (76%) of the 84 participants were women, 31 (37%) had fallen at least once during the two months before data collection, and 41 (49%) used assistive device outdoors (Table 1).

All items in the ABC were filled in by all participants. One person failed to perform the TUG^cognitive^ due to language difficulties that became apparent only in the stressful test situation. Two persons failed to perform the Four Square Step test due to the need to use rolling walker. 

**Figure 1:**
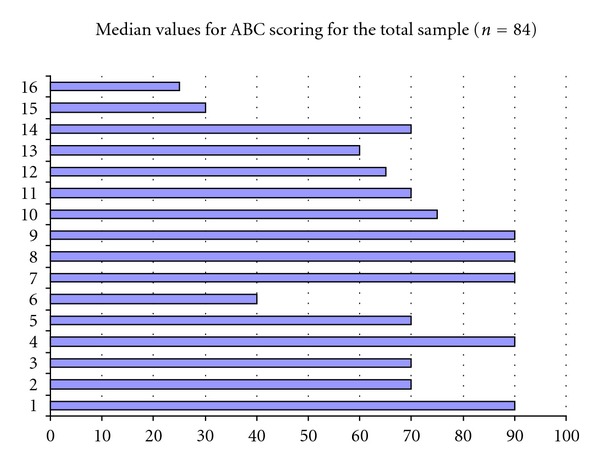
1 walk around the house, 2 walk up and down stairs, 3 bend over and pick up a slipper from the front of a closet floor, 4 reach for a small can off a shelf at eye level, 5 stand on your tip toes and reach for something above your head, 6 stand on a chair and reach for something, 7 sweep the floor, 8 walk outside the house to a car parked in the driveway, 9 get into or out of a car, 10 walk across a parking lot to the mall, 11 walk up and down a ramp, 12 walk in a crowded mall where people rapidly walk past you, 13 are bumped into by people as you walk through the mall, 14 step onto or off an escalator while holding onto a railing, 15 step onto or off an escalator while holding onto parcels, and 16 walk on icy sidewalks.

The median score for the ABC was 66 (IQR 45–80) for the total sample (Figure 1). A wide variation of ratings was present for the items. There were floor and ceiling effects for each ABC item except item 1 “walk around the house” (min 10 : max 100) but not for the ABC score (min 23.1; max 96.88). The most challenging activities were standing on a chair and reaching for something, stepping onto or off an escalator without hand support, and walking on icy sidewalks. The items most frequently rated with “complete confidence” were walking around the house, reaching for a small can on a shelf at eye level, sweeping the floor, walking outside the house to a car parked in the driveway, getting into and out of a car, and walking across a parking lot to the mall. Still, only one-third of the sample felt completely confident for each of these activities individually. 

The internal consistency was high at *α* = 0.95. The separate items ranged from 0.64 (item 9) to 0.81 (item 10). Cronbach's alpha did not change if item 9 “getting in or out of a car” was removed, suggesting that this item adds little value. The interitem correlations presented a median correlation at 0.60 (IQR 0.52–0.66). The highest interitem correlation (0.83) was found between the two activities connected with the lowest rated balance confidence (walk on icy sidewalks; step onto or off an escalator without hand support) (Table 2). No specific patterns were found by grouping the items as mainly reflecting anticipatory postural adjustments (stairs, ramp, escalator and icy sidewalks), postural responses (being bumped into, walk on icy sidewalks) or as stability limits/verticality (bending, reaching). The interitem correlations were not higher by grouping items as indoor or outdoor activities. 

The fallers (≥1 fall) performed worse than the non-fallers for all outcome measures with the exception of TUG^cog^ (Table 3).

As hypothesized, the concurrent convergent validity was overall moderate to good (0.50 to −0.75; Table 4), with the lowest correlation found for the TUG^cog^ and the highest for the MSWS-12. There were no statistically significant differences in balance confidence using the ABC between those reporting one or more falls during the two months prior to the testing procedure compared to those reporting no falls. Median score was 68 (IQR 53–84) among fallers (≥fall) and 64 (IQR 40–75) among non-fallers, giving a difference in ABC total score of only 4 points. However, a significant difference (*P* ≤ 0.02) was found between non-fallers and the multiple fallers (median ABC score 48; IQR 38–69). A statistically significant difference at *P* ≤ 0.001 was also found in ABC scoring between those reporting using assistive device outdoors or not. Lower balance confidence was reported by users (median score 56; IQR 38–68) compared to nonusers (median score 74; IQR 64–88). 

There were no statistically significant differences between men and women for the ABC score (*P* = 0.77). The median score was 67 (IQR 47–75) for men and 66 (IQR 45–83) for women. Statistical differences between men and women were also absent for the other outcome measures. 

## 4. Discussion

The validity of the ABC when used on PwMS was further strengthened in the present study. The concurrent convergent validity between the ABC and the other outcome measures was moderate to good, as hypothesized, and the internal consistency remained high at *α* = 0.95. Balance confidence is clearly related to balance function although the concepts differ somewhat. Higher correlations were found in the present study for both the Dynamic Gait Index (*r* = 0.62) and Timed Up and Go (*r* = −0.61), compared to those previously reported for PwMS (*r* = 0.54 and *r* = −0.38, resp.) in an Italian sample (*n* = 51) [28]. Perceptions of limitations in walking and balance confidence were closely related. Both the ABC and the 12-item MS Walking Scale are self-rating scales and incorporate standing and walking activities though the 12-item MS Walking Scale is focused on limitations in walking rather than balance confidence during activities. 

The inclusion of both fallers and non-fallers is a strength when validating balance confidence. Those reporting one fall the past two months and non-fallers showed no difference in balance confidence according to the ABC, contradicting previous results where fallers scored on average 24 points lower than non-fallers [28]. In that study, the non-fallers scored a mean of 61, while in the present study they scored a median of 68 and a mean of 66, though the samples otherwise seem comparable in many ways. The fallers in the present study were somewhat older and used walking devices more frequently both indoors and outdoors, compared to the non-fallers. However, the ABC was able to statistically significantly discriminate between those reporting two falls or more and non-fallers. Furthermore, the ABC discriminated between those reported who need to use assistive walking device outdoors and nonusers, and the need to use walking device presumably reflects a more pronounced disability. Both the use of assistive walking device and a higher impact of MS have been reported to be associated with falling. The instructions of the ABC reads that if a person normally uses walking device or support, the items should be rated as if one were using this. Still, the use of assistive walking device does not seem to fully compensate for the mobility impairment since balance confidence remains reported as more limited. The transfer of having limited balance confidence to the use of walking device may be considered as ecological validity of the ABC. 

Balance confidence did, as expected, not differ between men and women. Men and women did differ in terms of age (men: 55 years, IQR 38–60; women: 49 years, IQR 42–58), which may have interfered with the comparison. These results differ from those reported by Peterson et al. [14] and is most likely explained by their use of an older sample of PwMS (mean age 63.6; SD = 9.3.). Future, larger studies could investigate both possible gender differences in balance confidence and any gender dependence for different activities.

One strength of the ABC is that it incorporates everyday activities both indoors and outdoors. It is notable that even activities such as walking around the house, reaching, and sweeping were considered challenging by two-thirds of the sample, especially since 56% of the participants were currently working or studying. The restrictions on participation in society are clear, given that two-thirds of the PwMS in this study were not completely confident in being able to walk across a parking lot and get into or out of a car. Walking on icy sidewalks is both geographically and seasonally dependent. The instructions for the ABC state that if you have not performed the activity recently, you should imagine how confident you would be if performing it. The disadvantage of this is that it may be difficult to rate the actual ability, due to the length of time since last having performed the activity and the progressive path of MS. 

The internal validity of the study was strengthened by using physiotherapists who had received suitable training to acquaint them with the test procedure and standardized protocols. The physical tests were performed before the self-rating procedure, which may have affected the participants' perception, but none of the rated items were identical to the physical outcome measures. Perception of one's ability to perform an activity may differ from one's actual ability; for example, studies have shown both overestimation (43–47%) and underestimation (36–37%) of maximum walking distance [46, 47]. The present study used outcome measures that required the participants to actually perform activities. 

The choice of outcome measures for validation was primarily focused on stability in gait and standing activities, but also included anticipatory postural adjustment [13]. Since the ABC incorporates items related to reactive postural response (crowded shopping malls and being bumped into), tests measuring compensatory stepping could have been included. A fall could be regarded as the ultimate consequence of balance impairment, and hence as a perfect gold standard. Unfortunately, falls do not reflect less-pronounced balance problems which still may have a severe impact on everyday life.

Balance confidence is considered as a personal factor using the ICF model [48] while the TUG, TUG^cog^, 25TW, FSST, DGI, and CST are measures at the level of activity. The use of assistive walking device is an environmental factor, facilitating activity and participation. The ICF model can be used to structure outcome measures and findings and their mutual relation. Future research within this area is warranted. 

The ABC was easily administered to the studied sample and took approximately 5 minutes to fill in. The standardized instructions of the questionnaires gave sufficient information to the participants, and the scales might as well have been filled in without the presence of trained personnel. The scales can be administered by any caregiver. The patient could also fill in the questionnaires at home and bring them to an appointment in clinical praxis, for time efficiency. 

### 4.1. Study Limitations

The sample in the present study was restricted to PwMS interested in participating in balance exercise, which may have affected the generalizability. The sample is considered representative for type of MS, but the proportion of women was larger than that expected in the population. Even if more women than men are diagnosed with MS, the ratio in this study was 4 : 1. The sample was also restricted to include only those who still had the ability to walk at least 100 m. A decision based upon the use of measures for testing convergent validity. Balance impairment is surely also an issue for those with more explicit walking limitation, and the results of the present or previous [28] study cannot be generalized to more severely disabled people (EDSS > 6). The use of self-rating scales may also be problematic for those with severe cognitive disability. Finally, another limitation is the reliance on retrospectively collected data on previous falls since a correlation between daily reported and retrospectively reported falls has been reported at *r* = 0.82 [12].

## 5. Conclusions

Lack of balance confidence is present in many everyday activities for PwMS and should be taken into consideration along with the physical and environmental components. This study adds information of the validity of the ABC to measure balance confidence in PwMS. This study could not show any differences between men and women in balance confidence. The ABC did discriminate multiple fallers from non-fallers.

## Figures and Tables

**Table 1:** Demographic data. Medians and interquartile ranges (25–75%) presented for age and time since diagnose, mean for walking speed, otherwise frequency and percentage.

Variable	Total *n* = 84	Women *n* = 64	Men *n* = 20	Faller ≥ 1 *n* = 31	Nonfaller *n* = 53
Faller	31 (37)	20 (45)	11 (55)	—	—
Age	51 (42–58)	49 (42–58)	55 (38–60)	53 (45–60)	48 (40–58)
Time since diagnose	12 (6–18)	12 (7–18)	12 (2–18)	12 (6–19)	10 (6–17)
Walking speed (m/s)	1.17	1.13	1.30	1.11	1.21
MS type					
RR	54 (64)	44 (69)	10 (50)	19 (61)	35 (66)
SP	26 (31)	19 (30)	7 (35)	10 (32)	16 (30)
PP	4 (5)	1 (2)	3 (15)	2 (6)	2 (4)
Walking device					
indoors	15 (18)	11 (17)	4 (20)	8 (26)	7 (13)
outdoors	41 (49)	32 (50)	9 (45)	21 (68)	20 (38)
Work/study	47 (66)	35 (65)	12 (75)	16 (52)	31 (58)

RR: relapsing remitting; SP: secondary progressive; PP: primary progressive.

**Table 2:** Interitem correlations.

Spearmancorrelationcoefficients, N=84																
Prob > |*r*| under H0: Rho = 0																
	ABC1	ABC2	ABC3	ABC4	ABC5	ABC6	ABC7	ABC8	ABC9	ABC10	ABC11	ABC12	ABC13	ABC14	ABC15	ABC16
ABC1	1.00000	0.66569	0.63454	0.65059	0.57478	0.48893	0.66190	0.71276	0.52921	0.63457	0.67226	0.51262	0.57378	0.49827	0.46291	0.44971
ABC1		<.0001	<.0001	<.0001	<.0001	<.0001	<.0001	<.0001	<.0001	<.0001	<.0001	<.0001	<.0001	<.0001	<.0001	<.0001

ABC2	0.66569	1.00000	0.76454	0.61200	0.63539	0.59604	0.60740	0.50754	0.45716	0.63826	0.62254	0.66650	0.74176	0.52627	0.53233	0.58021
ABC2	<.0001		<.0001	<.0001	<.0001	<.0001	<.0001	<.0001	<.0001	<.0001	<.0001	<.0001	<.0001	<.0001	<.0001	<.0001

ABC3	0.63454	0.76454	1.00000	0.68223	0.74292	0.65222	0.71503	0.49266	0.59159	0.64500	0.59889	0.68480	0.67928	0.61980	0.55049	0.57786
ABC3	<.0001	<.0001		<.0001	<.0001	<.0001	<.0001	<.0001	<.0001	<.0001	<.0001	<.0001	<.0001	<.0001	<.0001	<.0001

ABC4	0.65059	0.61200	0.68223	1.00000	0.64855	0.40478	0.70234	0.65758	0.61205	0.67180	0.61768	0.53229	0.52339	0.55293	0.39940	0.35929
ABC4	<.0001	<.0001	<.0001		<.0001	0.0001	<.0001	<.0001	<.0001	<.0001	<.0001	<.0001	<.0001	<.0001	0.0002	0.0008

ABC5	0.57478	0.63539	0.74292	0.64855	1.00000	0.65969	0.68367	0.45428	0.56326	0.64610	0.52297	0.64615	0.60878	0.58051	0.54853	0.57701
ABC5	<.0001	<.0001	<.0001	<.0001		<.0001	<.0001	<.0001	<.0001	<.0001	<.0001	<.0001	<.0001	<.0001	<.0001	<.0001

ABC6	0.48893	0.59604	0.65222	0.40478	0.65969	1.00000	0.57963	0.31152	0.29992	0.45867	0.48783	0.56582	0.58485	0.51175	0.64559	0.58798
ABC6	<.0001	<.0001	<.0001	0.0001	<.0001		<.0001	0.0039	0.0056	<.0001	<.0001	<.0001	<.0001	<.0001	<.0001	<.0001

ABC7	0.66190	0.60740	0.71503	0.70234	0.68367	0.57963	1.00000	0.63499	0.51993	0.66100	0.58961	0.60406	0.64206	0.61778	0.61159	0.56848
ABC7	<.0001	<.0001	<.0001	<.0001	<.0001	<.0001		<.0001	<.0001	<.0001	<.0001	<.0001	<.0001	<.0001	<.0001	<.0001

ABC8	0.71276	0.50754	0.49266	0.65758	0.45428	0.31152	0.63499	1.00000	0.63621	0.78180	0.66300	0.50706	0.51220	0.43048	0.48793	0.43333
ABC8	<.0001	<.0001	<.0001	<.0001	<.0001	0.0039	<.0001		<.0001	<.0001	<.0001	<.0001	<.0001	<.0001	<.0001	<.0001

ABC9	0.52921	0.45716	0.59159	0.61205	0.56326	0.29992	0.51993	0.63621	1.00000	0.72451	0.51351	0.54734	0.43886	0.51719	0.49294	0.47524
ABC9	<.0001	<.0001	<.0001	<.0001	<.0001	0.0056	<.0001	<.0001		<.0001	<.0001	<.0001	<.0001	<.0001	<.0001	<.0001

ABC10	0.63457	0.63826	0.64500	0.67180	0.64610	0.45867	0.66100	0.78180	0.72451	1.00000	0.67564	0.72939	0.68519	0.58741	0.63158	0.59860
ABC10	<.0001	<.0001	<.0001	<.0001	<.0001	<.0001	<.0001	<.0001	<.0001		<.0001	<.0001	<.0001	<.0001	<.0001	<.0001

ABC11	0.67226	0.62254	0.59889	0.61768	0.52297	0.48783	0.58961	0.66300	0.51351	0.67564	1.00000	0.73705	0.64892	0.42631	0.51779	0.50330
ABC11	<.0001	<.0001	<.0001	<.0001	<.0001	<.0001	<.0001	<.0001	<.0001	<.0001		<.0001	<.0001	<.0001	<.0001	<.0001

ABC12	0.51262	0.66650	0.68480	0.53229	0.64615	0.56582	0.60406	0.50706	0.54734	0.72939	0.73705	1.00000	0.80383	0.53447	0.65441	0.70346
ABC12	<.0001	<.0001	<.0001	<.0001	<.0001	<.0001	<.0001	<.0001	<.0001	<.0001	<.0001		<.0001	<.0001	<.0001	<.0001

ABC13	0.57378	0.74176	0.67928	0.52339	0.60878	0.58485	0.64206	0.51220	0.43886	0.68519	0.64892	0.80383	1.00000	0.54795	0.66559	0.70481
ABC13	<.0001	<.0001	<.0001	<.0001	<.0001	<.0001	<.0001	<.0001	<.0001	<.0001	<.0001	<.0001		<.0001	<.0001	<.0001

ABC14	0.49827	0.52627	0.61980	0.55293	0.58051	0.51175	0.61778	0.43048	0.51719	0.58741	0.42631	0.53447	0.54795	1.00000	0.80014	0.62482
ABC14	<.0001	<.0001	<.0001	<.0001	<.0001	<.0001	<.0001	<.0001	<.0001	<.0001	<.0001	<.0001	<.0001		<.0001	<.0001

ABC15	0.46291	0.53233	0.55049	0.39940	0.54853	0.64559	0.61159	0.48793	0.49294	0.63158	0.51779	0.65441	0.66559	0.80014	1.00000	0.83108
ABC15	<.0001	<.0001	<.0001	0.0002	<.0001	<.0001	<.0001	<.0001	<.0001	<.0001	<.0001	<.0001	<.0001	<.0001		<.0001

ABC16	0.44971	0.58021	0.57786	0.35929	0.57701	0.58798	0.56848	0.43333	0.47524	0.59860	0.50330	0.70346	0.70481	0.62482	0.83108	1.00000
ABC16	<.0001	<.0001	<.0001	0.0008	<.0001	<.0001	<.0001	<.0001	<.0001	<.0001	<.0001	<.0001	<.0001	<.0001	<.0001	

**Table 3:** Mean and SD of the outcome measures for the total sample and the subgroups non-fallers and fallers.

	Total		Fallers (≥1)		Nonfallers	
	*n* = 84		*n* = 31		*n* = 53	
	Mean	SD	Mean	SD	Mean	SD
TUG	11.83	6.02	12.52	5.57	11.43	6.28
TUG^cog^ (*n* = 83)	14.32	7.07	13.94	4.91	14.53	8.08
25TW	6.51	3.24	6.85	2.67	6.32	3.55
FSST (*n* = 82)	17.29	13.00	18.18	12.34	16.74	13.47
DGI	17.08	4.62	15.06	3.98	18.26	4.60
CST	34.11	11.35	38.20	12.59	31.71	9.91
MSWS-12	51.44	25.26	59.61	20.40	46.66	26.75
ABC	63.74	20.42	59.78	64.38	66.06	20.75

**Table 4:** Convergent validity between the ABC and the other tests, *n* = 84 unless other reported.

Variable	Correlation coefficient	*P* value
Timed Up and Go test	−0.61	<.0001
Timed Up and Go^cognitive^ test (*n* = 83)	−0.50	<.0001
Timed 25-foot walk test	−0.63	<.0001
Four Square Step test (*n* = 82)	−0.59	<.0001
Dynamic Gait Index	0.62	<.0001
Timed Chair Stand test	−0.61	<.0001
12-item MS Walking Scale	−0.75	<.0001
